# Supplementary figures and images for: RDC complex executes a dynamic piRNA program during Drosophila spermatogenesis to safeguard male fertility
Source: PLoS Genet. 2021 Sep 2;17(9):e1009591. doi: 10.1371/journal.pgen.1009591 (PMC8412364; doi:10.1371/journal.pgen.1009591)

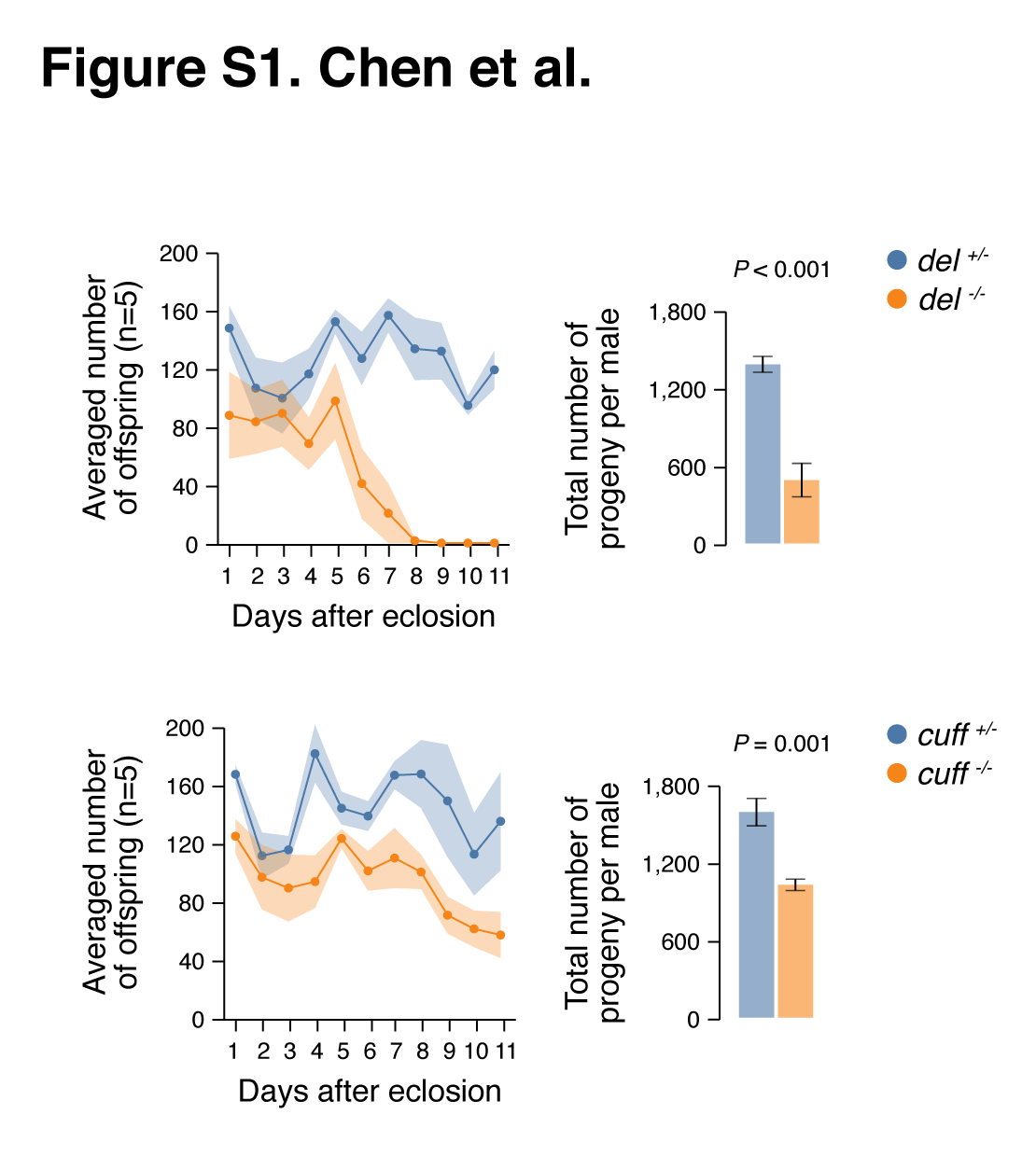

Supplement: S1 Fig — Compromised fertility of del (top) and cuff (bottom) mutant males. Sperm exhaustion test of delHN/WK or cuffWM25/QQ37 mutant (orange) and respective heterozygous sibling control (blue) males. Left: averaged numbers of offspring per male 1–11 days after eclosion (n = 5). Right: total number of progeny per male after mating for 11 days. Shaded areas and error bars display standard error. P value from unpaired t-test. (TIF) [file pgen.1009591.s001.tif]

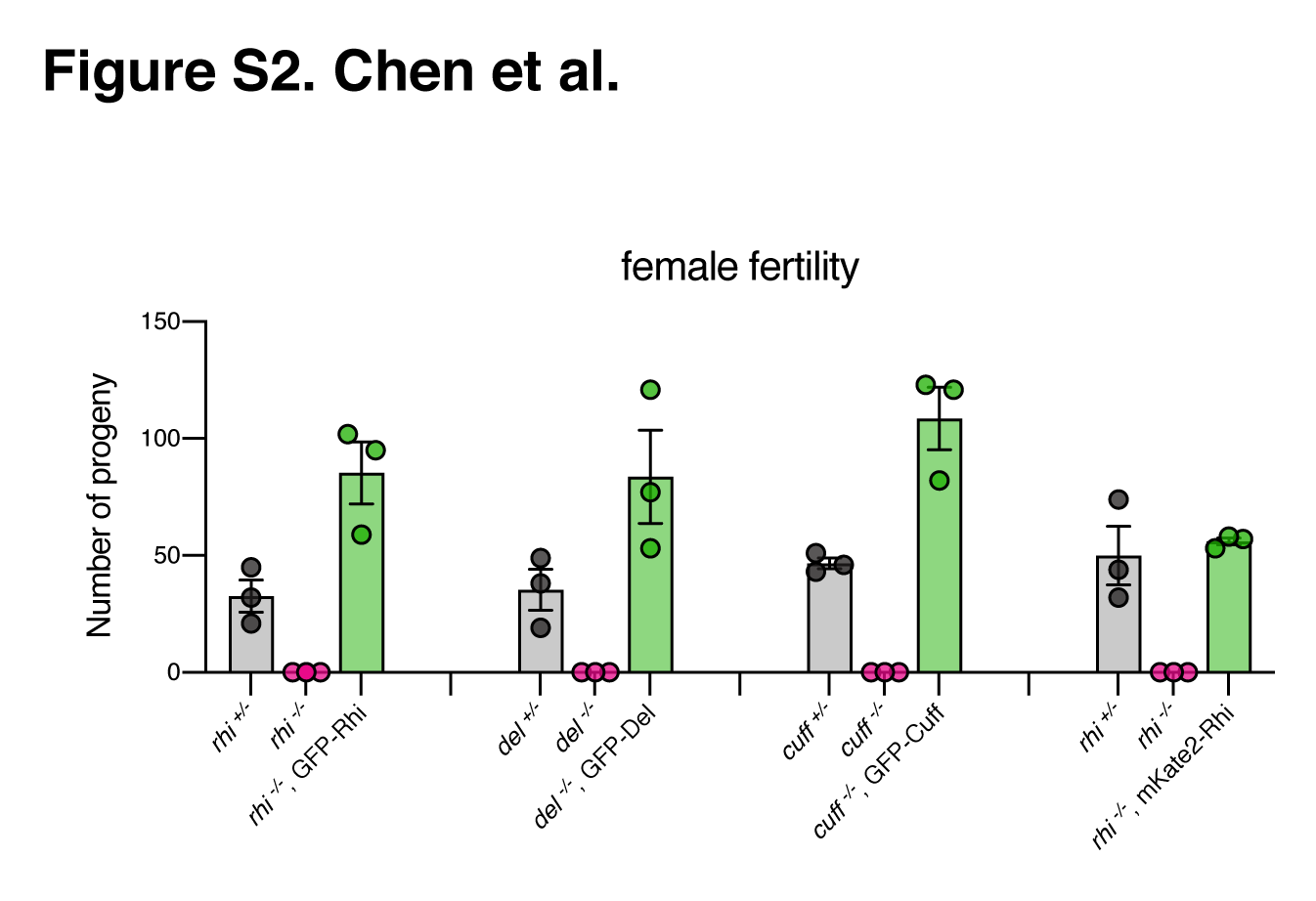

Supplement: S2 Fig — GFP-tagged Rhi, Del and Cuff transgenes as well as mKate2-tagged Rhi transgene driven by a putative rhi promoter fully rescue the female sterility of respective mutations. (TIF) [file pgen.1009591.s002.tif]

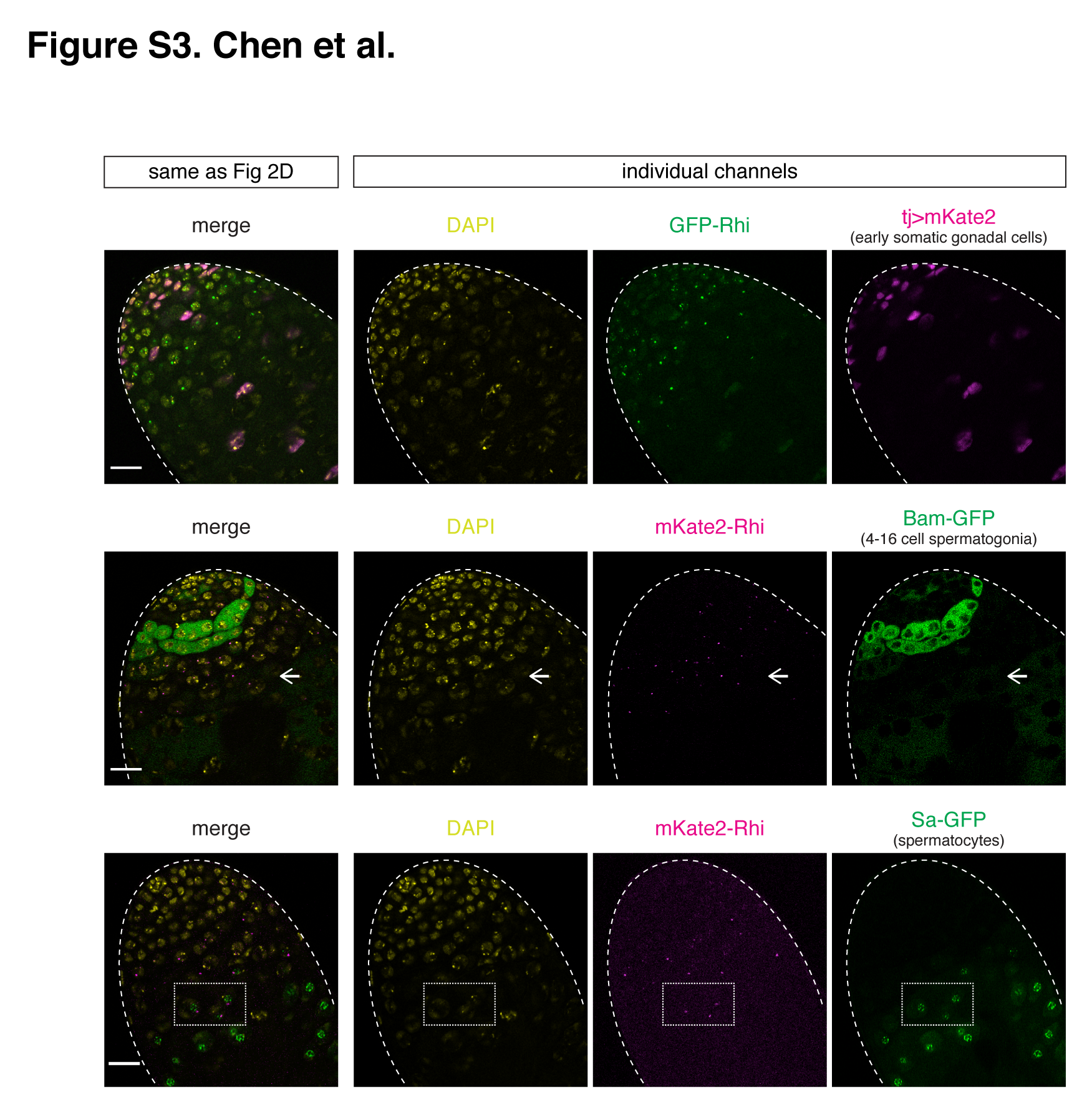

Supplement: S3 Fig — Individual channels of images shown in Fig 2D. (TIF) [file pgen.1009591.s003.tif]

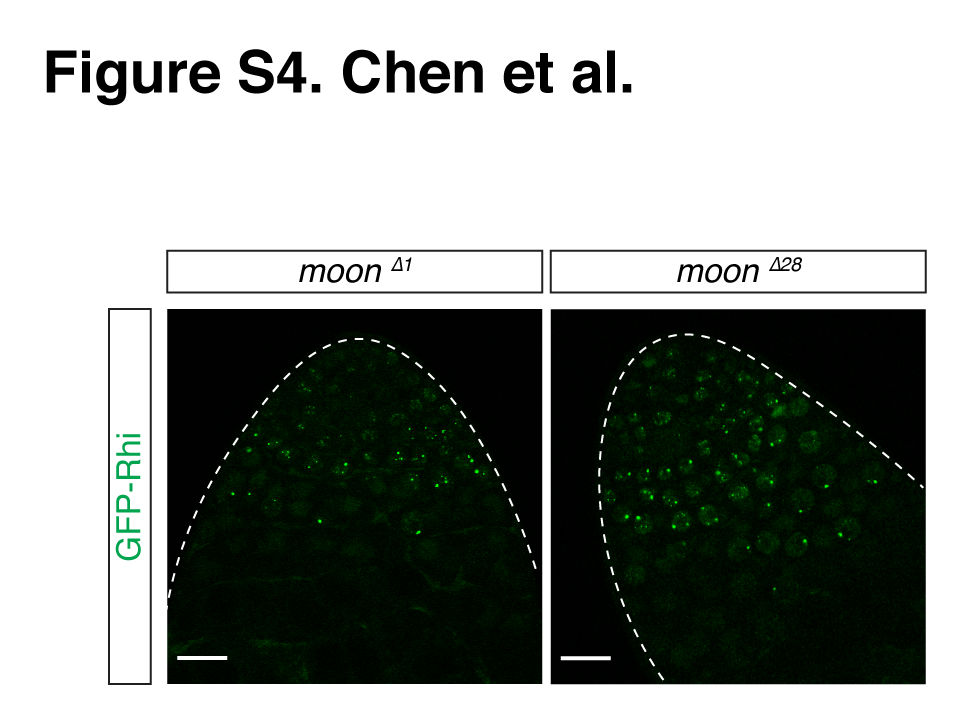

Supplement: S4 Fig — Confocal images of apical tips of testes expressing GFP-Rhi, in moonΔ1 and moonΔ28 mutant backgrounds. Note that moon is X-linked, so XY males only have one copy of moon and trans-heterozygous mutant cannot be generated. (TIF) [file pgen.1009591.s004.tif]

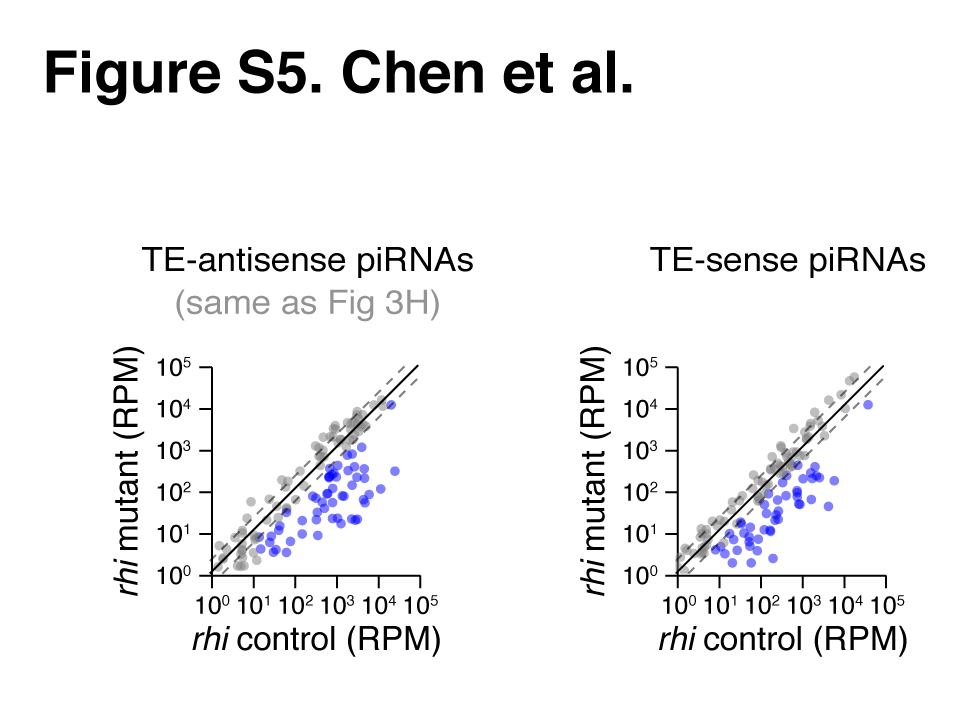

Supplement: S5 Fig — Loss of TE-mapping piRNAs in testes of rhi mutants. Scatter plot showing expression of TE-antisense (left) and TE-sense (right) piRNAs in rhi2/KG mutant versus heterozygous control testes. piRNAs that show ≥2-fold reduction (FDR < 0.05) and ≥10 RPM average expression levels are marked in blue. Shown are averages of two biological replicates. (TIF) [file pgen.1009591.s005.tif]
