# Supplementary material for: RDC complex executes a dynamic piRNA program during Drosophila spermatogenesis to safeguard male fertility
Source: PLoS Genet. 2021 Sep 2;17(9):e1009591. doi: 10.1371/journal.pgen.1009591 (PMC8412364; doi:10.1371/journal.pgen.1009591)
Supplement: Attachment — Submitted filename: Aravin (sub PLoS Genet 2020).pdf [file pgen.1009591.s006.pdf]

PGENETICS-D-20-01652
RDC complex executes a dynamic piRNA program during Drosophila spermatogenesis to safeguard male fertility
Peiwei Chen, Yicheng Luo, Alexei A. Aravin

In this manuscript, Aravin and coworkers set out to answer the question, do piRNA source loci in *Drosophila melanogaster* males use the same non-canonical mechanism of transcription to generate piRNA precursor RNAs as used in ovaries? They use a combination of genetics, qualitative and quantitative fluorescence microscopy, qPCR, and high-throughput small RNA, poly(A)+ RNA, and ChIP sequencing, to show that the piRNA clusters expressed in fly testes do indeed rely on the "RDC," a complex comprising the proteins Rhino (an HP1 variant), Deadlock, and Cutoff. In ovaries, the RDC marks all convergently transcribed (dual-strand) piRNA clusters. The protein Moonshiner then links the RDC to the canonical RNA polymerase II initiation machinery, allowing promoterless transcriptional initiation. Intriguingly, transcription of the testis-specific locus that lead to the discovery of piRNAs nearly 20 years ago, *Su(Ste)*, does not require these proteins, suggesting that, like piRNAs in some arthropods and mammals, *Su(Ste)* is canonically transcribed by RNA polymerase II, with initiation beginning through conventional, albeit convergent, promoters. In general, the manuscript is easy to read and the experiments well done. I am confident that with a few additional experiments and revision of text, the manuscript will be one *PLoS Genetics* should be proud to publish.

Major Concerns

(1) Beginning on page 6, experiments are presented that use GFP-tagged Rhino, Deadlock, Cuff, or Moonshiner. Are these actually transgenes? The manuscript completely elides this point, leaving readers to wonder if the GFP has been introduced into the endogenous locus using CRISPR/Cas9. These need to be discussed in much more detail, establishing why it is appropriate to infer endogenous protein expression and localization. Are the transgenes expressed at endogenous levels? The promoters appear to be from the endogenous locus, but how do the authors know they have correctly identified the promoters? Do they recapitulate the authentic sites and levels of expression in ovaries? Most importantly, does each transgene rescue the corresponding null mutation, especially when subjected to the sperm exhaustion test?

(2) Figure 3H: why do some antisense piRNAs increase in the absence of Rhino? What clusters or transposons do they come from? Does Rhino bind them? How are their precursors transcribed? Are these transcripts found in wild-type or are they unique to the RDC mutants?

(3) Why was poly(A)+ RNA used to quantify TE expression? The methods indicate that this was to avoid sequencing cluster transcripts (this should be validated in the manuscript by showing the abundance of cluster transcripts compared to TE transcripts). But if TEs outside clusters are bound by Rhino in testis as they are in ovary, then aren't the TE transcripts in wild-type also poly(A)−? Are uni-strand cluster transcripts readily detected in poly(A)+ RNA?

(4) The relative change in abundance of piRNAs and long RNAs is thresholded at a twofold change. But there is no mention of significance being used as a threshold. What FDR value was used? What *p*-values, corrected for false-discovery rate, are associated with the changes of the highlighted transposon families? As the manuscript now stands, the reader is left wondering if the changes might be large but non-significant.

(5) The authors claim that "Loss of RDC complex causes DNA damage and germ cell death in testis," but no cell death marker is used to substantiate this. Currently, how can the data distinguish between cell death and arrest of cell division?

(6) Is the decrease in piRNAs from a transposon family correlated in a statistically significant way with the increase in poly(A)+ RNA from the same family?

(7) The most exciting finding (at least to me) is that *Su(Ste)* is Rhino-independent. Are there Mod-ENCODE ChIP or published data that reveal the chromatin marks present on *Su(Ste)* in testis? Are the *Su(Ste)* loci euchromatic? Poly(A)+? Spliced?

(8) On page 10 the authors note that they "observed a correlation between Rhi binding and piRNA levels." I could not find the corresponding scatterplot, correlation coefficient, or *p*-value. Did I overlook it? Figure 4F does not report a correlation coefficient or its significance.

(9) The only part of this paper that I found hard to believe was the argument that the "dot" of Rhino was on the *AT-chX* locus alone. I think I am being fair in summarizing the authors' data as showing that the dot is not on the Y chromosome and is not *38C*. Couldn't the dot also be multiple loci clustering together? The claim that there is a single dot is not supported by showing a single focal plane; the entire z-stack needs to reconstructed and shown in a single image. This is especially important as the fly satellite DNAs from all chromosomes cluster together in spermatogonia and other mitotic cells into nuclear structures known as chromocenters (see, for example, Fig. 1C in Jagannathan et al., *eLife* 2019). In early spermatocytes, this single cluster separates into multiple chromocenters organized according to satellite sequence; at this stage the X, Y, and 4th chromosomes cluster into a single "dot." As a repetitive locus, perhaps *AT-chX* is part of the chromocenter? Does the dot also contain the protein D1 or Prod (Proliferation disrupter)? D1 is associated with the X/Y/4 chromocenter.

Minor Points

(1) Fig. 1A: “Days after eclosion” would be more standard English and would actually fit better as an x-axis label than “Day number after eclosion.”
(2) Page 4: missing from the description of how piRNA precursors are generated is the requirement for Maelstrom to suppress canonical transcription from the promoters of individual transposons embedded in piRNA clusters.
(3) Page 5ff: the plural of *progeny* in English is *progeny*. It’s a weird language.
(4) Page 7: *in-between*, not *in between*.
(5) Page 6: Sa protein is introduced for the first time without any reference to the unabbreviated gene name, which I assume is *spermocyte arrest*.”
(6) Page 8: doesn’t the *copia* reporter establish that *rhi* is required for transcriptional silencing. It might be worth mentioning this.
(7) Are the lines in Fig. 4F generated by Loess smoothing? What do they tell us?

(8) Page 14: I don’t understand why cluster piRNAs and the RDC form a feed-forward loop. The architecture of an FFL is (Alon, *Nat Rev Genet* 2007):

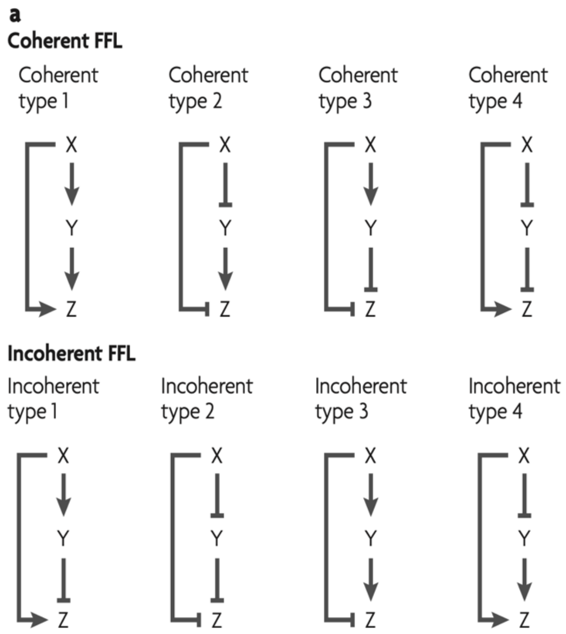

Perhaps the authors mean a single or double positive-feedback loop?
(9) It should be easy to test informatically whether piRNAs corresponding to transposons present in *H17* are deposited in the embryo, since unfertilized egg piRNAs have been sequenced.
(10) Are all sequencing data in the manuscript presented as the mean of two replicates? This needs to be added to the figure legend or methods.
