# Supplementary material for: RDC complex executes a dynamic piRNA program during Drosophila spermatogenesis to safeguard male fertility
Source: PLoS Genet. 2021 Sep 2;17(9):e1009591. doi: 10.1371/journal.pgen.1009591 (PMC8412364; doi:10.1371/journal.pgen.1009591)
Supplement: Attachment — Submitted filename: 20210421_PlosGenet_ResponsesToReviewers.pdf [file pgen.1009591.s007.pdf]

Responses to reviewers

PGENETICS-D-20-01652

RDC complex executes a dynamic piRNA program during Drosophila spermatogenesis to safeguard male fertility

Peiwei Chen, Yicheng Luo, Alexei A. Aravin

We are very grateful to all three reviewers for their valuable criticism and suggestions. We performed new experiments and analyses and made changes to the text of the paper to address the comments. Below, we provide our point-by-point response (shown in blue) to the comments of each reviewer.

Reviewer #1: In this manuscript, Aravin and coworkers set out to answer the question, do piRNA source loci in Drosophila melanogaster males use the same non-canonical mechanism of transcription to generate piRNA precursor RNAs as used in ovaries? They use a combination of genetics, qualitative and quantitative fluorescence microscopy, qPCR, and high-throughput small RNA, poly(A)+ RNA, and ChIP sequencing, to show that the piRNA clusters expressed in fly testes do indeed rely on the "RDC," a complex comprising the proteins Rhino (an HP1 variant), Deadlock, and Cutoff. In ovaries, the RDC marks all convergently transcribed (dual-strand) piRNA clusters. The protein Moonshiner then links the RDC to the canonical RNA polymerase II initiation machinery, allowing promoterless transcriptional initiation. Intriguingly, transcription of the testis-specific locus that lead to the discovery of piRNAs nearly 20 years ago, Su(Ste), does not require these proteins, suggesting that, like piRNAs in some arthropods and mammals, Su(Ste) is canonically transcribed by RNA polymerase II, with initiation beginning through conventional, albeit convergent, promoters. In general, the manuscript is easy to read and the experiments well done. I am confident that with a few additional experiments and revision of text, the manuscript will be one PLoS Genetics should be proud to publish.

<u>Major Concerns</u>

(1) Beginning on page 6, experiments are presented that use GFP-tagged Rhino, Deadlock, Cuff, or Moonshiner. Are these actually transgenes? The manuscript completely elides this point, leaving readers to wonder if the GFP has been introduced into the endogenous locus using CRISPR/Cas9. These need to be discussed in much more detail, establishing why it is appropriate to infer endogenous protein expression and localization. Are the transgenes expressed at endogenous levels? The

promoters appear to be from the endogenous locus, but how do the authors know they have correctly identified the promoters? Do they recapitulate the authentic sites and levels of expression in ovaries? Most importantly, does each transgene rescue the corresponding null mutation, especially when subjected to the sperm exhaustion test?

We have added additional description and characterization of fluorescently tagged Rhino, Deadlock, Cutoff and Moonshiner flies in the text on page 6 and 7, in the figure legends and the method section. We also added a new supplementary figure that demonstrates rescue of corresponding mutations.

GFP-tagged Rhino, Deadlock, Cutoff and Moonshiner are transgenes published previously by the Brennecke lab (Mohn et al., 2014; Andersen et al., 2017), where each of the GFP-tagged factors is expressed under the control of its native regulatory region and promoter. The transgenes were constructed by transferring the gene and its extended flanking regions (several Kb upstream and downstream of each gene) using recombineering from BAC genomic clones. Thus, transgenes are expected to provide the native expression pattern for each protein. Rescue of the female sterility of *moon*$^{-/-}$ by GFP-Moon was reported previously (Andersen et al., 2017), while the authors did not examine rescue for other transgenes. Now we have performed female fertility tests to show that GFP-Rhi, GFP-Del and GFP-Cuff fully rescue the female sterility seen in *rhi*$^{-/-}$, *del*$^{-/-}$ and *cuff*$^{-/-}$, respectively. We included these results in the revised manuscript (new Fig S2).

mKate2-Rhi is a transgene we made using a strategy described by the Theurkauf lab (Zhang et al., 2014). This transgene contains a ~2 Kb region upstream of the Rhi start codon, providing native regulation of Rhi expression. This region includes a pronounced RNA pol II peak near the *rhi* TSS, consistent with the inclusion of a *bona fide* promoter and extended upstream region. Importantly, Zhang et al. demonstrated that expressing the transgene by this putative promoter region provides functional rescue of *rhi* mutations, as it reduces the high splicing efficiency of certain transcripts observed in *rhi* mutants to a level comparable to wildtype controls. Now we have verified that the mKate2-Rhi transgene also fully rescues the female sterility of *rhi*$^{-/-}$ (new Fig S2).

Regarding expression levels, it is important to note that Rhi, Del and Cuff form a complex and co-localize in distinct nuclear puncta when expressed at physiological levels. In contrast, strong overexpression of one component will lead to a diffused localization in nuclei as observed in early germline of *nos-Gal4>UASp-GFP-Rhi* testes shown in Fig 6C (bottom right). The lack of such a diffused pattern in Rhi, Del and Cuff transgenes we used argues against an expression level higher than what is physiologically relevant. Furthermore, genome-wide localization patterns of GFP-Rhi and GFP-Cuff have been analyzed previously by ChIP-seq (Le Thomas et al., 2014). Both tagged proteins have been shown to bind the same loci as native proteins do, recapitulating binding profiles obtained by ChIP-seq

with antibodies against endogenous Rhi and Cuff (Klattenhoff et al., 2009; Mohn et al., 2014; Zhang et al., 2014). We hope these explanations justify our choice to use these transgenes to study RDC.

(2) Figure 3H: why do some antisense piRNAs increase in the absence of Rhino? What clusters or transposons do they come from? Does Rhino bind them? How are their precursors transcribed? Are these transcripts found in wild-type or are they unique to the RDC mutants?

In Fig 3I (previously Fig 3H), there are 22 TE-antisense piRNAs (out of 87) that showed an increase in *rhi* mutant testes (FDR < 0.01). Note that all of these are also present in control testes and the observed increases are mild (<4-fold), in stark contrast to many TE-antisense piRNAs that showed drastic (>4-fold) reductions (Fig 3H, previously Fig 3G). Many of the TEs that show mild piRNA increase (14 out of 22, e.g., *ZAM*, *mdg1*, *IDEFIX* and *tabor*) are TEs that can be found in uni-strand clusters *flamenco* and *20A,* which are free of Rhi binding and transcribed in a Rhi-independent, canonical manner. Thus, since we normalized expression levels to the total number of reads in each library, their mild up-regulation likely reflects an increase in the relative abundance of these piRNAs in the total piRNA pool caused by the global loss of piRNAs produced from dual-strand clusters in the *rhi* mutant (rather than an increase in their cellular concentrations). We are unsure why piRNAs against the remaining 8 TEs also increased mildly, but 7 of them showed opposite changes in the two sexes (i.e., they increased in testis but decreased in ovary upon loss of *rhi*), a question that awaits future studies. Nonetheless, we would like to emphasize that this observation does not affect our claim that many TE-targeting piRNAs showed dramatic reductions following the loss of *rhi* in testis, as shown in Fig 3H.

(3) Why was poly(A)+ RNA used to quantify TE expression? The methods indicate that this was to avoid sequencing cluster transcripts (this should be validated in the manuscript by showing the abundance of cluster transcripts compared to TE transcripts). But if TEs outside clusters are bound by Rhino in testis as they are in ovary, then aren't the TE transcripts in wild-type also poly(A)−? Are uni-strand cluster transcripts readily detected in poly(A)+ RNA?

We agree with the reviewer that, in wildtype flies, transcripts derived from TEs outside clusters but bound by Rhi likely lack polyA tails. Such transcripts, which are believed to be initiated from multiple internal sites rather than proper promoters (Mohn et al., 2014; Andersen et al., 2017), are channeled into the piRNA processing machinery but likely excluded from translation. Our goal was to see if the absence of Rhi causes an increase in translationally-competent TE transcripts that have polyA tails, so we deliberately wanted to exclude non-canonical transcripts. We expect canonically transcribed TE

transcripts that are translationally competent to be polyadenylated. In contrast, piRNA precursor transcripts from dual-strand clusters were shown to lack polyA tails (Mohn et al., 2014; Chen et al., 2016). It was also shown that, upon disruption of the RDC complex in the ovary, the level of non-canonically transcribed transcripts from dual-strand clusters decreases, while expression of canonical TE transcripts increases (Klattenhoff et al., 2009; Mohn et al., 2014). Using total RNA (or rRNA-depleted RNA), one cannot easily distinguish these two types of transcripts. Therefore, we used polyA-selection to exclude non-canonical transcripts. We have now explicitly mentioned the goal to exclude non-canonical transcripts from individual TEs in the Methods section.

Our polyA+ RNA-seq libraries contain only a few reads from uni-strand clusters. One possible reason might be that uni-strand clusters 20A and flamenco are only active at the apical tip of testis. Detecting stage-specific, lowly expressed transcripts might require using *bam* mutant testis that arrests spermatogenesis at the spermatogonia-to-spermatocyte transition.

(4) The relative change in abundance of piRNAs and long RNAs is thresholded at a twofold change. But there is no mention of significance being used as a threshold. What FDR value was used? What p-values, corrected for false-discovery rate, are associated with the changes of the highlighted transposon families? As the manuscript now stands, the reader is left wondering if the changes might be large but non-significant.

We thank the reviewer for pointing this out. We have added additional cutoff of FDR < 0.05 on top of ≥2-fold change for both TE mRNA and piRNA quantification. We have updated Fig 3G and 3H so that only TEs with adjusted p-value < 0.05 are colored (though Fig 3H remains unchanged), and we have edited figure legends and text on page 8 and 9 to indicate the significance cutoff employed.

(5) The authors claim that "Loss of RDC complex causes DNA damage and germ cell death in testis," but no cell death marker is used to substantiate this. Currently, how can the data distinguish between cell death and arrest of cell division?

We attempted to image cell death directly using a dye that stains apoptotic cells (LysoTracker). However, we noticed that heterozygous control and wildtype testes were frequently stained as well, reflecting a high basal cell death rate in the male germline. High basal cell death rate has been previously described (Yacobi-Sharon et al. 2013), presumably as a result of male germline quality control mechanisms. Also, cell death markers can only stain cells that are dying at the moment but does not provide any information on cells that have died in the past. Meanwhile, we observed

prominent gaps between Vasa-positive germline cysts in mutant testes but never in controls (Fig 3A, middle two rows). These gaps are likely a result of germ cells that have died and cleared from the tissue. Importantly, we observed some mutant testes completely depleted of early germ cells but, nevertheless, have mature sperm in the seminal vesicle, suggesting that spermatogenesis proceeded without arrest of cell division. In the revised manuscript, we added new images of seminal vesicles filled with mature sperm in mutant testes that lack early male germline altogether (new Fig 3D) to formally demonstrate that there is no arrest in cell division or spermatogenesis. We have described this in the text on page 8.

(6) Is the decrease in piRNAs from a transposon family correlated in a statistically significant way with the increase in poly(A)+ RNA from the same family?

We have now analyzed the correlation between fold increase of polyA+ transposon expression and fold reduction of transposon-antisense piRNAs for every transposon family in testis upon mutating *rhi*. From this analysis, we found a moderate and statistically significant correlation between log-transformed values of the two (Spearman's $\rho = 0.41$, $P = 7.3 \times 10^{-5}$; Pearson's $\rho = 0.46$, $P = 8.4 \times 10^{-6}$). This has now been described in the text on page 9.

(7) The most exciting finding (at least to me) is that Su(Ste) is Rhino-independent. Are there Mod-ENCODE ChIP or published data that reveal the chromatin marks present on Su(Ste) in testis? Are the Su(Ste) loci euchromatic? Poly(A)+? Spliced?

We are glad that the reviewer shares our excitement about this finding. The Drosophila Y chromosome, where Su(Ste) resides, is believed to be entirely heterochromatic. Unfortunately, modENCODE does not include testis ChIP-seq datasets, and no H3K9me2/3 ChIP-seq has been published for testis either. According to Aravin et al. 2001 (Fig 1), sense transcripts of Su(Ste) are spliced and polyadenylated (as expected, since it shares the splice site and polyA site with Ste genes), suggesting their canonical transcription and co-transcriptional processing. This is consistent with the Su(Ste) locus lacking RDC, which suppresses splicing and polyadenylation. We have now described this in the Discussion on page 15.

(8) On page 10 the authors note that they “observed a correlation between Rhi binding and piRNA levels.” I could not find the corresponding scatterplot, correlation coefficient, or p-value. Did I overlook it? Figure 4F does not report a correlation coefficient or its significance.

In the original manuscript, we did local regression but indeed did not calculate the correlation coefficient. We have now computed it and confirmed that, for Rhi-dependent loci, there is a strong correlation between Rhi binding and piRNA production (Spearman’s $\rho = 0.97$; Pearson’s $\rho = 0.99$). We have reported this in the text on page 10.

(9) The only part of this paper that I found hard to believe was the argument that the “dot” of Rhino was on the AT-chX locus alone. I think I am being fair in summarizing the authors’ data as showing that the dot is not on the Y chromosome and is not 38C. Couldn’t the dot also be multiple loci clustering together? The claim that there is a single dot is not supported by showing a single focal plane; the entire z-stack needs to reconstructed and shown in a single image. This is especially important as the fly satellite DNAs from all chromosomes cluster together in spermatogonia and other mitotic cells into nuclear structures known as chromocenters (see, for example, Fig. 1C in Jagannathan et al., eLife 2019). In early spersmatocytes, this single cluster separates into multiple chromocenters organized according to satellite sequence; at this stage the X, Y, and 4th chromosomes cluster into a single “dot.” As a repetitive locus, perhaps ATchX is part of the chromocenter? Does the dot also contain the protein D1 or Prod (Proliferation disrupter)? D1 is associated with the X/Y/4 chromocenter.

We thank the reviewer for raising these points. We performed additional experiments to examine the nature of the Rhino “dot” and reported our new findings in the revised manuscript. First, we analyzed entire z-stacks to be certain that there is only a single Rhi dot in the nuclei of early spermatocytes. We have presented a maximum-intensity projection image covering the depth of an entire nucleus in Fig 6A (right). Second, we explored if the Rhi dot corresponds to chromocenters of several chromosomes clustered together by imagining Rhi together with Prod and D1 proteins that bind satellite DNA. We found that D1 which binds AATAT satDNA on chrX/Y/4 does not co-localize with the Rhi dot (new Fig 6F). In contrast to D1, expression of Prod which binds AATAACATAG satDNA on chr2/3 ceases at spermatogonia-to-spermatocyte transition (Jagannathan et al., 2019, Fig 3 – Fig S1). Therefore, Prod protein is absent in spermatocytes when the single Rhi dot is formed. In addition, we observed that the single Rhi dot often persists till when individual chromosome territories are formed in spermatocyte nuclei. In these nuclei, the connection between non-homologous chromosomes have mostly dissolved as suggested by ≥4 GFP-Cid dots that mark centromeres (new Fig 6G). These new results suggest that

the Rhi dot does not correspond to chromocenters of several chromosomes clustered together. We have described these new findings on page 12.

Minor Points

(1) Fig. 1A: "Days after eclosion" would be more standard English and would actually fit better as an x-axis label than "Day number after eclosion."

We thank the reviewer for this suggestion; we have changed it as suggested.

(2) Page 4: missing from the description of how piRNA precursors are generated is the requirement for Maelstrom to suppress canonical transcription from the promoters of individual transposons embedded in piRNA clusters.

We have now cited this work.

(3) Page 5ff: the plural of progeny in English is progeny. It's a weird language.

We thank the reviewer for this correction. We corrected it throughout the manuscript.

(4) Page 7: in-between, not in between.

Corrected.

(5) Page 6: Sa protein is introduced for the first time without any reference to the unabbreviated gene name, which I assume is spermocyte arrest."

We have clarified this; the unabbreviated gene name is indeed "spermatocyte arrest".

(6) Page 8: doesn't the copia reporter establish that rhi is required for transcriptional silencing. It might be worth mentioning this.

The reporter consists of the *copia* LTR and the *lacZ* gene. The transcription start site is located in the middle of the *copia* LTR (see Kalmykova et al., 2005 PMID 15817569 Fig 2A), so part of the *copia* LTR is transcribed along with *lacZ* as a fusion transcript. As a result, the reporter can be subject to transcriptional or post-transcriptional silencing by piRNA/PIWI complexes, or both. Since Rhi is required for piRNA production in testis, loss of piRNAs in *rhi* mutants will indirectly disrupt both transcriptional and post-transcriptional silencing of targets. Thus, we cannot exclude the possibility of post-transcriptional silencing of the *copia* reporter and decided not to make a claim about the mode of repression.

(7) Are the lines in Fig. 4F generated by Loess smoothing? What do they tell us?

We performed local regression (LOESS) to obtain a curve that describes the relationship between two variables of interest. Its advantage is that it does not require prior assumptions of the curve shape. As shown in Fig 4E (previously Fig 4F), local regression of Rhi-dependent loci revealed two regions showing quasi-linear relationship with a near-zero slope for $x < 4$ but a positive slope for $x > 4$. This suggested that for Rhi-dependent loci producing sufficient piRNAs, an increase in Rhi binding likely yields an increase in piRNA production, consistent with the strong correlation we observed between Rhi binding and piRNA production (as mentioned above for Major Concern #8).

(8) Page 14: I don't understand why cluster piRNAs and the RDC form a feed-forward loop. The architecture of an FFL is (Alon, Nat Rev Genet 2007): Perhaps the authors mean a single or double positive-feedback loop?

We thank the reviewer for pointing this mistake out and for referencing Uri Alon's work. We have now corrected "feed-forward loop" to "positive-feedback loop".

(9) It should be easy to test informatically whether piRNAs corresponding to transposons present in H17 are deposited in the embryo, since unfertilized egg piRNAs have been sequenced.

We have looked at piRNA data from unfertilized eggs and found that piRNAs corresponding to TEs present at the *petrel* (previously named as *h17*) locus are indeed deposited into the embryo. For example, *roo* piRNAs are the most abundant ones compared to any other TE among piRNAs deposited into the embryo (Brennecke et al., 2008), and several *roo* fragments can be found at the *petrel* locus. In fact, *roo* sequences occupy 5.8% of the total *petrel* cluster length, making it the third most prevalent TE by length (after *IDEFIX* 12.8% and *ninja* 6.9%). piRNAs against other TEs present at the *petrel* cluster can also be found in unfertilized eggs. We have added this information to the Discussion section of the revised manuscript.

(10) Are all sequencing data in the manuscript presented as the mean of two replicates? This needs to be added to the figure legend or methods.

All sequencing data is presented as the mean of two replicates, except the track view of piRNA-seq and ChIP-seq in Fig 4F and 5B, where the profile of a representative replicate is shown. We have now explicitly described this in all figure legends and in the methods.

Reviewer #2: In this manuscript, Chen et al. explores the function of RDC (Rhino-Deadlock-Cutoff) complex in Drosophila testis. RDC complex mediates transcription of piRNA cluster, and has been believed to function only in female germline. However, Chen et al. provide a convincing evidence that RDC complex has a function in male germline as well, by showing compromised fertility of male RDC mutants. They further characterize targets of RDC complex in the testis, in comparison to ovary, providing insights into the regulation and biogenesis of piRNA in Drosophila testis. This is an important study to clarify the function of piRNA pathway components in the testis via thorough analysis, and is a significant contribution to the field.

I have no major issues with this manuscript, with only minor suggestions as following.

-Fig3A, ‘no germline’ is not entirely accurate. I see elongated sperm tails (as the haze of green background). It should be labeled as ‘early germ cell depletion’.
We thank the reviewer for this comment; we have now changed the label in Fig 3A to “early germ cell depletion” and edited the text description accordingly.

-The passage in the discussion ‘Also, after transition to spermatocytes, germ cells lose their ability to de-differentiate back to germline stem cells (Brawley and Matunis, 2004). It is thus tempting to propose that piRNA pathway contributes to the maintenance of cellular plasticity in early male germ cells (GSCs, gonialblasts and spermatogonia) by ensuring robust genome defense.’: there is no evidence to go against this statement, but it is generally thought that pre-meiotic S phase would prepare chromosome/chromatids to be compatible with meiosis (consecutive homolog segregation and sister segregation without intervening S phase), and therefore, it is highly unlikely that they can resume mitotic divisions. Therefore, the comments to suggest piRNA might maintain germ cell plasticity, merely basing on the expression pattern of piRNA, seems to be extrapolation. This is a discussion, so the author should be allowed to say almost anything they like, but I think this discussion might rather hurt authors for putting a rather careless discussion. So I am just pointing it out.

We thank the reviewer for pointing this out. We have deleted this passage, given its speculative nature, and replaced it with a different discussion.

Reviewer #3: This manuscript from Chen et al, revealed the role of Rhino and the RDC complex in piRNA biogenesis from dual strand piRNA clusters in testis all along spermatogenesis. The role of Rhino was already well characterized in piRNA biogenesis in ovaries but due to a lack of mutant phenotype in males was remain largely unexplored in testis. A detail examination of the male fertility by sperm exhaustion test revealed subfertility of the Rhino mutant males. A spatio temporal careful analysis indeed revealed that the RDC complex is not ovary-specific and assembled also in early spermatogenesis which is required for piRNA production and TE silencing. Surprisingly and interestingly the piRNA biogenesis of suppresser of stellate piRNAs which is expressed from a particular dual strand cluster is not Rhino dependent.

The manuscript is well written and fairly easy to follow. The data are mostly well presented and clearly explained. The main criticism is that all along the manuscript the authors conclude on the effect of a loss of the RDC complex while they investigate, most of the time, only the loss of Rhino. All the experiments presented in this paper (small RNA sequencing, copia LacZ staining, TE mRNA expression …) are not investigated in del or cuff mutant. Overall, this is an interesting paper that is suitable for publication in Plos Genetics, as long as the following minor criticisms can be addressed.

<u>Minor points</u>

Figure 1: The phenotype of the Rhino is very well described and it is clear that Rhino is required for male fertility. Are Deadlock and Cutoff required like Rhino for male fertility?

We have performed sperm exhaustion test for *deadlock* and *cutoff* mutant males and found that, similar to *rhino*, both mutants showed a significant decline in male fertility (n=5, $P \leq 0.001$). Thus, all three components of the RDC complex are required for maintaining normal male fertility. These new results are described in the text on page 5 and shown in the new Fig S1.

Figure 2B: Rhino is dispersed in the nuclei and not puncta anymore in del or cuff mutants. However, in del or cuff KD ovaries the protein Rhino is completely lost as demonstrated in the paper of ElMaghraby et all. The difference between testis and ovaries should be discussed.

We thank the reviewer for noticing this difference. However, observations of Rhi expression and localization upon del or cuff KD in ovary have been variable in the literature. Therefore, we are not sure if there is a significant difference between ovary and testis and decided not to discuss it. In Mohn et al. 2014 (PMID 24906153) Fig S4A, Rhi signal is weaker and dispersed but not completely lost in cuff KD. In Andersen et al. 2017 (PMID 28847004) Extended Data Fig 3g, Rhi signal is weaker and dispersed

but not completely lost in del KD. Both work came from the same group that published ElMaghraby et al. 2019. In addition, in Kneuss et al. 2019 (PMID 31416967) Fig 2A published by a different group, Rhi signal is also not completely lost in either del or cuff KD. Despite these variabilities, it is important to note that Rhi in ovary cannot form proper puncta or localize properly to dual-strand clusters when either Del or Cuff is perturbed (regardless of whether Rhi is dispersed or completely lost), consistent with our observation in testis. In fact, in both ovary and testis, each of the three components of the RDC complex relies on the simultaneous presence of the other two factors to form puncta, suggesting that it functions as an inter-dependent tripartite protein complex.

Figure 2D: Only the merge is presented. For a better visualization and interpretation an independent staining of each proteins should be added to this figure.

We have now shown individual channels in the new Fig S3.

Figure 2A: The expression of Moon appears to be more diffused in the nucleus in the GSC and then punctuated in the spermatogonia. It is not obvious that Moonshiner colocalize with Rhino in the GSC. It should be mentioned and discussed.

In general, localization of Moon appears to be more diffused in the nucleus than Rhino, as also observed in ovary according to Andersen et al. 2017 (PMID 28847004) Fig 2d and Extended Data Fig 3f. However, because expression and localization pattern of Moon in GSCs are clearly abolished in *rhi* mutants, we favor the idea that the dependence of Moon on an intact RDC complex spans from GSC to early spermatocyte, throughout its expression window in testis. We have explicitly discussed this point in the revised manuscript (page 7).

Only the antisense piRNAs which are the regulatory ones are presented. Since Rhino is involved in dual strand piRNA cluster expression it would be nice if the authors could also present the effect of a loss of Rhino on sense piRNAs.

We have now analyzed the profile of TE-sense piRNAs in *rhi* mutants and found a similar effect as seen for TE-antisense piRNAs. This result is shown in the new Fig S5 and described in the text on page 9.

From the experiment presented figure 3, the authors should conclude that the loss of Rhino instead of RDC complex leads to a reduction in the germ cell count and GSC population in testis since they have only investigated Rhino mutant.

To address the role of other RDC components, we have examined *del* and *cuff* mutant testes and found frequent depletion of all early germ cells (including GSCs), which were not observed in heterozygous sibling controls. These results, which demonstrate that all three components of RDC are required for proper spermatogenesis, are shown in the new Fig 3D and described in the text on page 8. We have also edited the text to only conclude on Rhi on page 8 until we described results of *del* and *cuff*.

The interpretation of the figure 4A is that Rhino and not the RDC complex is essential for piRNA production from a large fraction of piRNA clusters in male germline.

When *rhi* is mutated, Del and Cuff either de-localize or are de-stabilized and thus are no longer able to localize properly to their specific genomic target loci. For this reason, we are unable to confidently attribute the phenotypes observed in *rhi* mutants to either *rhi* loss itself or the indirect perturbation of Del and Cuff proteins. To further explore the role of other RDC components, we have characterized additional phenotypes in testis lacking either *del* or *cuff.* We observed compromised male fertility (as mentioned above, new Fig S1), frequent depletion of early germline (as mentioned above, new Fig 3D), DNA double-strand breaks as evidenced by γ-H2Av staining (new Fig 3E) and *copia* derepression as shown using the *copia-lacZ* reporter (new Fig 3F), in both *del* and *cuff* mutants, reminiscent of those observed in *rhi* mutants. Comparable phenotypes when mutating either component of the RDC complex, as well as the inability to assign *rhi* mutant phenotypes to Rhi itself rather than indirect effects on Del and Cuff, suggest that the observed phenotypes are caused by disruption of RDC complex function. Nevertheless, we have now softened the claim and edited the text to only formally conclude about the importance of Rhi on page 10 in the revised manuscript.

Again, the conclusion of ChIP experiments should be that Rhino and not the RDC complex binds the chromatin and ensures the expression of Dual strand piRNA-clusters.

We agree with the reviewer that we do not have direct evidence that Del and Cuff localize to the exact same places on the genome as Rhi, so we have softened the claim and edited the text on page 10 to only formally conclude on Rhi localization.

Do the authors know whether TEs contained in the 38C piRNA cluster are TEs which are specifically expressed in testis when they are derepressed ? What is the size of the 38C cluster?

We thank the reviewer for raising this interesting point, which we have addressed in a separate paper we recently posted on BioRxiv (doi: 10.1101/2020.08.25.266585). In that work, we have analyzed the sex bias of piRNA cluster expression and the sex bias in transposon content of cluster that are differentially expressed in the two sexes (Fig 5 in that manuscript). Indeed, as suggested by the reviewer, 38C is enriched for transposons that show stronger derepression in the testis than in the ovary upon disruption of piRNA pathway. 38C is about 65Kb long.

To explain the specific localization at one single locus of Rhino, the authors proposed that since the germ cells are diploid it is plausible that this locus resides on one of the two sets chromosomes. It is well described in drosophila cells that the two sets of autosomes are pairs together and form only one dot in the nucleus in FISH. In ovaries the pairing originates immediately after the stem cell stage. This pairing occurs well before the initiation of meiosis and, strikingly, continues through the several mitotic divisions preceding meiosis. Is it not the case in male germ cells?

We thank the review for pointing this out. Homolog pairing is best described in the Drosophila soma. For early male germline (up to early spermatocyte), we are only aware of a few papers like Vazquez et al. 2002 (PMID 12225662) and the review McKee et al. 2012 (PMID 23087836). It appears that homologs pair only in half (~50%) of spermatogonia and very early spermatocytes, according to Fig 1 of Vazquez et al. 2002, reflecting a high degree of variability in homolog pairing from cell to cell. We have observed one bright Rhi dot at these stages in almost all nuclei, pointing to the possibility that it is linked to a sex chromosome. We took a candidate-based approach to test possible co-localization of the Rhi protein and individual loci in question, and we were lucky to identify AT-chX, a locus on the X chromosome, that co-localizes with the one bright Rhi dot. To clear the confusion, we have now cited the reference and edited the text to reformulate our rationale on page 11.

To prove the specificity of the situ HCR experiment performed to detect nascent transcripts of the 38C locus the experiment should also be performed in a Rhino mutant genetic background.

We have now performed *in situ* HCR for 38C nascent transcripts in *rhi* mutant and heterozygous sibling control testes. As expected, we observed signal in control but not in *rhi* mutant testes, confirming its specificity. These results are shown in the new Fig 6D and described in the text on page 12.
